# Body mass index and subsequent fracture risk: a meta-analysis to update FRAX

**DOI:** 10.1093/jbmr/zjaf091

**Published:** 2025-08-08

**Authors:** Nicholas C Harvey, Helena Johansson, Eugene V McCloskey, Enwu Liu, Kristina E Åkesson, Fred A Anderson, Rafael Azagra-Ledesma, Cecilie L Bager, Charlotte Beaudart, Heike A Bischoff-Ferrari, Emmanuel Biver, Olivier Bruyère, Jane A Cauley, Jacqueline R Center, Roland Chapurlat, Claus Christiansen, Cyrus Cooper, Carolyn J Crandall, Steven R Cummings, José A P da Silva, Bess Dawson-Hughes, Adolfo Diez-Perez, Alyssa B Dufour, John A Eisman, Petra J M Elders, Serge Ferrari, Yuki Fujita, Saeko Fujiwara, Claus-Christian Glüer, Inbal Goldshtein, David Goltzman, Vilmundur Gudnason, Jill Hall, Didier Hans, Mari Hoff, Rosemary J Hollick, Martijn Huisman, Masayuki Iki, Sophia Ish-Shalom, Graeme Jones, Magnus K Karlsson, Sundeep Khosla, Douglas P Kiel, Woon-Puay Koh, Fjorda Koromani, Mark A Kotowicz, Heikki Kröger, Timothy Kwok, Olivier Lamy, Arnulf Langhammer, Bagher Larijani, Kurt Lippuner, Fiona E A McGuigan, Dan Mellström, Thomas Merlijn, Tuan V Nguyen, Anna Nordström, Peter Nordström, Terence W O’Neill, Barbara Obermayer-Pietsch, Claes Ohlsson, Eric S Orwoll, Julie A Pasco, Fernando Rivadeneira, Berit Schei, Anne-Marie Schott, Eric J Shiroma, Kristin Siggeirsdottir, Eleanor M Simonsick, Elisabeth Sornay-Rendu, Reijo Sund, Karin M A Swart, Pawel Szulc, Junko Tamaki, David J Torgerson, Natasja M van Schoor, Tjeerd P van Staa, Joan Vila, Nicholas J Wareham, Nicole C Wright, Noriko Yoshimura, M Carola Zillikens, Marta Zwart, Liesbeth Vandenput, Mattias Lorentzon, William D Leslie, John A Kanis

**Affiliations:** MRC Lifecourse Epidemiology Centre, University of Southampton, Southampton, SO16 6YD, United Kingdom; NIHR Southampton Biomedical Research Centre, University of Southampton and University Hospital Southampton NHS Foundation Trust, Southampton, SO16 6YD, United Kingdom; Centre for Metabolic Bone Diseases, University of Sheffield, Sheffield, United Kingdom; Centre for Metabolic Bone Diseases, University of Sheffield, Sheffield, United Kingdom; MRC Versus Arthritis Centre for Integrated Research in Musculoskeletal Ageing, Mellanby Centre for Musculoskeletal Research, University of Sheffield, Sheffield, United Kingdom; South Australian Health and Medical Research Institute (SAHMRI), Adelaide, SA, Australia; Clinical and Molecular Osteoporosis Research Unit, Department of Clinical Sciences, Lund University, Lund, Sweden; Department of Orthopedics, Skåne University Hospital, Malmö, Sweden; GLOW Coordinating Center, Center for Outcomes Research, University of Massachusetts Medical School, Worcester, MA, United States; Department of Medicine, Autonomous University of Barcelona, Barcelona, Spain; Docencia Atenció Primària, Metropolitana Nord, Institut Català de la Salut, Barcelona, Spain; GROIMAP/GROICAP (research groups), Unitat de Suport a la Recerca de Girona, Institut Universitari d’Investigació en Atenció Primària Jordi Gol, Girona, Spain; PRECIOSA-Fundación para la investigación, Barberà del Vallés, Barcelona, Spain; Nordic Bioscience A/S, Herlev, Denmark; Public Health Aging Research and Epidemiology (PHARE) Group, Research Unit in Clinical Pharmacology and Toxicology (URPC), NAmur Research Institute for LIfe Sciences (NARILIS), Faculty of Medicine, University of Namur, 5000 Namur, Belgium; Department of Aging Medicine, Felix Platter, University of Basel, 4055 Basel, Switzerland; Centre on Aging and Mobility, University of Zurich and City Hospital, Zurich, Switzerland; Division of Bone Diseases, Department of Medicine, Geneva University Hospitals and Faculty of Medicine, University of Geneva, Geneva, Switzerland; Research Unit in Public Health, Epidemiology and Health Economics, University of Liège, Liège, Belgium; Department of Epidemiology, School of Public Health, University of Pittsburgh, Pittsburgh, PA, United States; Garvan Institute of Medical Research, Sydney, NSW, Australia; St Vincent's Clinical School, School of Medicine and Health, University of New South Wales Sydney, Sydney, NSW, Australia; INSERM UMR 1033, Université Claude Bernard-Lyon1, Hôpital Edouard Herriot, Lyon, France; Nordic Bioscience A/S, Herlev, Denmark; MRC Lifecourse Epidemiology Centre, University of Southampton, Southampton, SO16 6YD, United Kingdom; NIHR Southampton Biomedical Research Centre, University of Southampton and University Hospital Southampton NHS Foundation Trust, Southampton, SO16 6YD, United Kingdom; NIHR Oxford Biomedical Research Centre, University of Oxford, Oxford, United Kingdom; Division of General Internal Medicine and Health Services Research, David Geffen School of Medicine, University of California, Los Angeles, CA, United States; San Francisco Coordinating Center, California Pacific Medical Center Research Institute, San Francisco, CA, United States; Centre for Innovative Biomedicine and Biotechnology, Faculty of Medicine, University of Coimbra, Coimbra, Portugal; Rheumatology Department, University Hospital and University of Coimbra, Coimbra, Portugal; Bone Metabolism Laboratory, Jean Mayer US Department of Agriculture Human Nutrition Research Center on Aging, Tufts University, Boston, MA, United States; Department of Internal Medicine, Hospital del Mar and CIBERFES, Autonomous University of Barcelona, Barcelona, Spain; Marcus Institute for Aging Research, Hebrew SeniorLife, Boston, MA, United States; Department of Medicine, Beth Israel Deaconess Medical Center and Harvard Medical School, Boston, MA, United States; St Vincent's Clinical School, School of Medicine and Health, University of New South Wales Sydney, Sydney, NSW, Australia; Skeletal Diseases Program, Garvan Institute of Medical Research, Sydney, NSW, Australia; School of Medicine Sydney, University of Notre Dame Australia, Sydney, NSW, Australia; Department of General Practice, Amsterdam UMC, location AMC, Amsterdam Public Health Research Institute, Amsterdam, The Netherlands; Division of Bone Diseases, Department of Medicine, Geneva University Hospitals and Faculty of Medicine, University of Geneva, Geneva, Switzerland; Department of Hygiene and Public Health, Faculty of Medicine, Kansai Medical University, Osaka, Japan; Department of Pharmacy, Yasuda Women’s University, Hiroshima, Japan; Section Biomedical Imaging, Molecular Imaging North Competence Center, Department of Radiology and Neuroradiology, University Medical Center Schleswig-Holstein Kiel, Kiel University, Kiel, Germany; Maccabitech Institute of Research and Innovation, Maccabi Healthcare Services, Tel Aviv, Israel; Department of Epidemiology and Preventive Medicine, Sackler Faculty of Medicine, School of Public Health, Tel Aviv University, Tel Aviv, Israel; Department of Medicine, McGill University and McGill University Health Centre, Montreal, QC, Canada; Icelandic Heart Association, Kopavogur, Iceland; University of Iceland, Reykjavik, Iceland; MRC Centre for Reproductive Health, University of Edinburgh, Edinburgh, United Kingdom; Interdisciplinary Centre of Bone Diseases, Bone and Joint Department, Lausanne University Hospital (CHUV) and University of Lausanne, Lausanne, Switzerland; Department of Neuromedicine and Movement Science, Norwegian University of Science and Technology, Trondheim, Norway; Department of Rheumatology, St. Olavs Hospital, Trondheim, Norway; Aberdeen Centre for Arthritis and Musculoskeletal Health, Epidemiology Group, University of Aberdeen, Aberdeen, United Kingdom; Department of Epidemiology and Data Science, Amsterdam Public Health Research Institute, VU University Medical Center, Amsterdam, The Netherlands; Department of Sociology, VU University, Amsterdam, The Netherlands; Department of Public Health, Kindai University Faculty of Medicine, Osaka, Japan; Endocrine Clinic, Elisha Hospital, Haifa, Israel; Menzies Institute for Medical Research, University of Tasmania, Hobart, TAS, Australia; Clinical and Molecular Osteoporosis Research Unit, Department of Clinical Sciences, Lund University, Lund, Sweden; Department of Orthopaedics, Skåne University Hospital, Malmö, Sweden; Robert and Arlene Kogod Center on Aging and Division of Endocrinology, Mayo Clinic College of Medicine, Mayo Clinic, Rochester, MN, United States; Department of Medicine, Beth Israel Deaconess Medical Center and Harvard Medical School, Boston, MA, United States; Marcus Institute for Aging Research, Hebrew Senior Life, Boston, MA, United States; Healthy Longevity Translational Research Programme, Yong Loo Lin School of Medicine, National University of Singapore, Singapore; Singapore Institute for Clinical Sciences, Agency for Science Technology and Research (A*STAR), Singapore; Department of Internal Medicine, Erasmus University Medical Center, Rotterdam, The Netherlands; Department of Radiology and Nuclear Medicine, Erasmus University Medical Center, Rotterdam, The Netherlands; Institute for Mental and Physical Health and Clinical Translation (IMPACT), Deakin University, Geelong, VIC, Australia; Barwon Health, Geelong, VIC, Australia; Department of Medicine-Western Health, The University of Melbourne, St Albans, VIC, Australia; Department of Orthopedics and Traumatology, Kuopio University Hospital, Kuopio, Finland; Kuopio Musculoskeletal Research Unit, University of Eastern Finland, Kuopio, Finland; Department of Medicine and Therapeutics, Faculty of Medicine, The Chinese University of Hong Kong, Hong Kong; Jockey Club Centre for Osteoporosis Care and Control, Faculty of Medicine, The Chinese University of Hong Kong, Hong Kong; Centre of Bone Diseases, Lausanne University Hospital, Lausanne, Switzerland; Service of Internal Medicine, Lausanne University Hospital, Lausanne, Switzerland; HUNT Research Centre, Department of Public Health and Nursing, Faculty of Medicine and Health Sciences, Norwegian University of Science and Technology, Trondheim, Norway; Levanger Hospital, Nord-Trøndelag Hospital Trust, Levanger, Norway; Endocrinology and Metabolism Research Center, Endocrinology and Metabolism Clinical Sciences Institute, Tehran University of Medical Sciences, Tehran, Iran; ARTORG Center for Biomedical Engineering Research, Faculty of Medicine, University of Bern, Bern, Switzerland; Clinical and Molecular Osteoporosis Research Unit, Department of Clinical Sciences, Lund University, Lund, Sweden; Geriatric Medicine, Department of Internal Medicine and Clinical Nutrition, Institute of Medicine, Sahlgrenska Academy, University of Gothenburg, Gothenburg, Sweden; Geriatric Medicine, Sahlgrenska University Hospital Mölndal, Mölndal, Sweden; Department of General Practice, Amsterdam UMC, location AMC, Amsterdam Public Health Research Institute, Amsterdam, The Netherlands; School of Biomedical Engineering, University of Technology Sydney, Sydney, NSW, Australia; School of Population Health, UNSW Medicine, UNSW Sydney, Kensington, NSW, Australia; Tam Anh Research Institute, Tam Anh Hospital, Ho Chi Minh City, Vietnam; School of Sport Sciences, UiT The Arctic University of Norway, Tromsø, Norway; Department of Health Sciences, Swedish Winter Sports Research Centre, Mid Sweden University, Östersund, Sweden; Department of Medical Sciences, Uppsala University, Uppsala, Sweden; Department of Public Health and Caring Sciences, Uppsala University, Uppsala, Sweden; National Institute for Health Research Manchester Biomedical Research Centre, Manchester University NHS Foundation Trust, Manchester Academic Health Science Centre, Manchester, United Kingdom; Centre for Epidemiology Versus Arthritis, University of Manchester, Manchester, United Kingdom; Division of Endocrinology and Diabetology, Department of Internal Medicine, Medical University Graz, Graz, Austria; Sahlgrenska Osteoporosis Centre, Department of Internal Medicine and Clinical Nutrition, Institute of Medicine, Sahlgrenska Academy, University of Gothenburg, Gothenburg, Sweden; Department of Drug Treatment, Sahlgrenska University Hospital, Gothenburg, Sweden; Department of Medicine, Oregon Health and Science University, Portland, OR, United States; Institute for Physical and Mental Health and Clinical Translation (IMPACT), Deakin University, Geelong, VIC, Australia; Department of Medicine-Western Health, The University of Melbourne, St Albans, VIC, Australia; Barwon Health, Geelong, VIC, Australia; Department of Epidemiology and Preventive Medicine, Monash University, Melbourne, VIC, Australia; Department of Internal Medicine, Erasmus University Medical Center, Rotterdam, The Netherlands; Department of Public Health and Nursing, Norwegian University of Science and Technology, Trondheim, Norway; Department of Obstetrics and Gynaecology, St Olavs Hospital, Trondheim, Norway; Université Claude Bernard Lyon 1, RESHAPE U INSERM U-1290, Lyon, France; Laboratory of Epidemiology and Population Sciences, National Institute on Aging, Baltimore, MD, United States; Icelandic Heart Association, Kopavogur, Iceland; Janus Rehabilitation, Reykjavik, Iceland; Translational Gerontology Branch, National Institute on Aging Intramural Research Program, Baltimore, MD, United States; INSERM UMR 1033, University of Lyon, Hôpital Edouard Herriot, Lyon, France; Kuopio Musculoskeletal Research Unit, University of Eastern Finland, Kuopio, Finland; Department of General Practice, Amsterdam UMC, location VUmc, Amsterdam Public Health Research Institute, Amsterdam, The Netherlands; PHARMO Institute for Drug Outcomes Research, Utrecht, The Netherlands; INSERM UMR 1033, University of Lyon, Hôpital Edouard Herriot, Lyon, France; Department of Hygiene and Public Health, Faculty of Medicine, Educational Foundation of Osaka Medical and Pharmaceutical University, Osaka, Japan; York Trials Unit, Department of Health Sciences, University of York, York, United Kingdom; Department of Epidemiology and Data Science, Amsterdam UMC location Vrije Universiteit, Amsterdam, The Netherlands; Amsterdam Public Health Research Institute, Aging and Later Life Program, Amsterdam, The Netherlands; Centre for Health Informatics, Faculty of Biology, Medicine and Health, School of Health Sciences, University of Manchester, Manchester, United Kingdom; Statistics Support Unit, Hospital del Mar Medical Research Institute, CIBER Epidemiology and Public Health (CIBERESP), Barcelona, Spain; MRC Epidemiology Unit, University of Cambridge, Cambridge, United Kingdom; Center for Health Outcomes, Implementation, and Community-Engaged Sciences, Tulane University, New Orleans, LA, United States; Department of Preventive Medicine for Locomotive Organ Disorders, The University of Tokyo Hospital, Tokyo, Japan; Department of Internal Medicine, Erasmus University Medical Center, Rotterdam, The Netherlands; PRECIOSA-Fundación para la investigación, Barberà del Vallés, Barcelona, Spain; Health Center Can Gibert del Pla, Catalan Institute of Health, Girona, Spain; Department of Medical Sciences, University of Girona, Girona, Spain; GROICAP (Research Group), Institut Universitari d’Investigació en Atenció Primària Jordi Gol, Girona, Spain; Sahlgrenska Osteoporosis Centre, Department of Internal Medicine and Clinical Nutrition, Institute of Medicine, Sahlgrenska Academy, University of Gothenburg, Gothenburg, Sweden; Sahlgrenska Osteoporosis Centre, Institute of Medicine, University of Gothenburg, Gothenburg, Sweden; Region Västra Götaland, Geriatric Medicine, Sahlgrenska University Hospital, Mölndal, Sweden; Department of Medicine, University of Manitoba, Winnipeg, MB, Canada; Centre for Metabolic Bone Diseases, University of Sheffield, Sheffield, United Kingdom

**Keywords:** BMI, meta-analysis, hip fracture, osteoporosis, epidemiology, major osteoporotic fracture, FRAX

## Abstract

The aim of this international meta-analysis was to quantify the predictive value of BMI for incident fracture and relationship of this risk with age, sex, follow-up time, and BMD. A total of 1 667 922 men and women from 32 countries (63 cohorts), followed for a total of 16.0 million person-years were studied. 293 325 had FN BMD measured (2.2 million person-years follow-up). An extended Poisson model in each cohort was used to investigate relationships between WHO-defined BMI categories (Underweight: <18.5 kg/m^2^; Normal: 18.5-24.9 kg/m^2^; Overweight: 25.0-29.9 kg/m^2^; Obese I: 30.0-34.9 kg/m^2^; Obese II: ≥35.0 kg/m^2^) and risk of incident osteoporotic, major osteoporotic and hip fracture (HF). Inverse-variance weighted β-coefficients were used to merge the cohort-specific results. For the subset with BMD available, in models adjusted for age and follow-up time, the hazard ratio (95% CI) for HF comparing underweight with normal weight was 2.35 (2.10-2.60) in women and for men was 2.45 (1.90-3.17). Hip fracture risk was lower in overweight and obese categories compared to normal weight [obese II vs normal: women 0.66 (0.55-0.80); men 0.91 (0.66-1.26)]. Further adjustment for FN BMD T-score attenuated the increased risk associated with underweight [underweight vs normal: women 1.69 (1.47-1.96); men 1.46 (1.00-2.13)]. In these models, the protective effects of overweight and obesity were attenuated, and in both sexes, the direction of association reversed to higher fracture risk in Obese II category [Obese II vs Normal: women 1.24 (0.97-1.58); men 1.70 (1.06-2.75)]. Results were similar for other fracture outcomes. Underweight is a risk factor for fracture in both men and women regardless of adjustment for BMD. However, while overweight/obesity appeared protective in base models, they became risk factors after additional adjustment for FN BMD, particularly in the Obese II category. This effect in the highest BMI categories was of greater magnitude in men than women. These results will inform the second iteration of FRAX®.

## Introduction

Body mass index (BMI), calculated as weight divided by height squared, is an accepted surrogate for adiposity in population studies and as a clinical risk factor in assessment for outcomes, such as myocardial infarction and fracture.^1^^,^^2^ Multiple studies have documented the complex relationships between fat mass, lean mass, and bone mineral density (BMD) in the determination of fracture risk.^3^^,^^4^ Obesity is associated with an increased risk of falling.^5^ Both low and high BMI have been associated with greater risk of fracture, but importantly, with different fracture sites at either end of the BMI spectrum: Findings from the Global Longitudinal study of Osteoporosis in Women (GLOW) suggested high risk of ankle and upper leg fractures with obesity,^6^ with similar results in the Spanish SIDIAP dataset.^7^ More recently, a Mendelian Randomization study demonstrated causal associations between low BMI and risk of forearm fracture.^8^ Consistent with these observations, in our previous meta-analysis of 398 610 women, mean age 63 years, low BMI was a risk factor for hip and all osteoporotic fractures, but appeared to be protective for lower leg/ankle fracture.^2^ In contrast, high BMI was associated with increased risk for humerus and elbow fractures. This relationship was dependent partly on BMD. Thus, after adjustment for FN BMD, high BMI remained a risk factor for upper arm fracture but also predicted increased risk of all osteoporotic fractures. BMI therefore conveys risk information for incident fracture, but with important considerations regarding the relationships with BMD and fracture site. However, the evidence base thus far principally focuses on women, with relationships in men, and indeed across ethnicities in either sex, still requiring detailed elucidation.

FRAX, currently available in 86 territories, is the most widely used fracture risk assessment tool and is incorporated into a large number of assessment guidelines,^9^ recommended by the Committee for Medicinal Products for Human Use (CHMP),^10^ and approved by the National Institute for Health and Care Excellence (NICE).^11^ The incorporation of BMI as an input variable for risk prediction was based on an earlier meta-analysis.^12^ Since then, many more prospectively studied cohorts have become available that have the potential to improve the accuracy of FRAX and understand better the potential influences of BMD, sex, and ethnicity in these relationships.^13^

The aim of the present study was to quantify magnitude of the risk relationship between BMI and incident fracture in an international setting, and to explore the dependence of this risk on age, sex, ethnicity, time since baseline assessment, and BMD.

## Materials and methods

Details of our initial systematic review undertaken to identify appropriate cohorts, and of the cohorts included, have been documented previously^13^ and are summarized in Table S1. This was registered in the PROSPERO international prospective register of systematic reviews (CRD42021227266), and followed the Preferred Reporting Items for Systematic Reviews (PRISMA) guidelines. In brief, we studied 1 667 922 men and women from 63 prospectively studied cohorts. A total of 57 cohorts included women (*n* = 1 127 206) and 40 cohorts included men (*n* = 540 716). Of these 293 325 men and women had femoral neck (FN) BMD measured, from 53 cohorts in 20 countries, with a total follow-up time of 2.2 million person-years.

### Baseline and outcome variables

BMI (kg/m^2^) was calculated from height and weight assessed at baseline. We considered incident fractures in the following categories: all, osteoporotic, major osteoporotic (MOF: clinical spine, distal forearm, hip, or humerus), and hip fracture (HF). For the purpose of this analysis, and related meta-analyses informing the next iteration of FRAX,^14–16^ we defined “osteoporotic fractures” on the basis of previously identified sites at which fractures were more common with increasing age and decreasing BMD, thus excluding fractures of the skull, face, hands, feet, ankle, and patella, as well as tibia and fibula fractures in men.^17^^,^^18^ This should not be taken to mean that anyone experiencing such a fracture has osteoporosis, merely that they appear more frequent in those with low BMD and/or higher age. No distinction was made according to trauma since both high- and low-trauma fractures show similar relationships with low BMD and future fracture risk.^19^^,^^20^ Details of fracture ascertainment and validation have been published previously.^13^

### Statistical methods

The risk of incident fracture, expressed as hazard ratio (HR) per 1 kg/m^2^ greater baseline BMI, was estimated using an extended Poisson model applied separately to each cohort (and also separately by sex for those cohorts with both men and women).^21^ Because of an embargo on data transfer, Cox regression was used on the Manitoba cohort. Covariates included current time since start of follow-up, current age, prior history of fracture, and BMD T-score at the FN. BMD was adjusted for manufacturer and T-scores were calculated from the NHANES III White female reference values.^13^ We categorized BMI according to WHO recommendations (Underweight: <18.5; Normal: 18.5-24.9 (referent); Overweight: 25.0-29.9; Obese I: 30.0-34.9; Obese II: ≥35.0 kg/m^2^)^22^ for analyses by category. We illustrated non-linear associations using spline models with nodes at 21, 25, and 33 kg/m^2^. Results of each cohort and the 2 sexes were weighted according to the variance and merged to determine the weighted means and standard deviations. The HR was equal to e^weighted mean of β^. A priori, a random effects model was used in the meta-analysis. Assessment of the effects of ethnicity was confined to those cohorts recording more than one ethnic group [Asian, Black, Hispanic, White and the Black, Asian and Minority Ethnic category (BAME)], comprising Health ABC, CAMOS, MrOS USA, LASA, WHI, SOF, Manitoba, and UK Biobank. Ascertainment of these data was via self-report. On the basis of these 8 cohorts there was sufficient data to undertake pairwise comparisons. Finally, we investigated associations between BMI and risk of death during follow-up.

## Results

Of 1 667 922 men and women studied in 32 countries, the average BMI was 27.2 kg/m^2^. At follow up, 151 473 men and women were identified as having a subsequent fracture of any kind; 125 135 were characterized as osteoporotic in men and women, 95 748 men and women sustained a MOF; 31 383 were HFs. The total follow-up time was 16.0 million-person years in men and women. BMD measurements were available in 17.6% (293325) of individuals. Table S1 summarizes key characteristics of the cohorts.

### Association between BMI and risk of incident fracture

**Figure 1:**
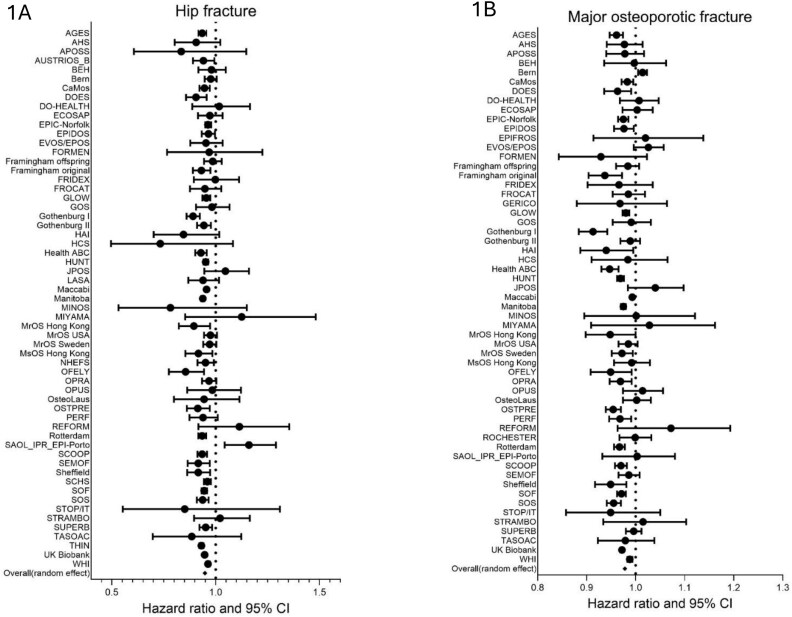
Forest plot showing effect size on hip fracture (HF) risk (left panel, A) and major osteoporotic fracture (right panel, B) per one unit greater BMI in men and women combined adjusted for age and time since baseline.

In continuous models, summarized in Table 1, after adjustment for age and time since baseline, greater BMI was associated with lower risk of incident fracture in both men and women, for example, HR (95% CI) for major osteoporotic fracture per unit greater BMI in women was 0.98 (0.97, 0.98); in men 0.98 (0.97, 0.98). Figure 1 documents these associations for HF and major osteoporotic fracture in men and women combined as Forest plots. Associations appeared similar by sex and were not materially different in the subset of cohorts in which BMD was measured. In this subset, after further adjustment for BMD, there was a reversal in the relationships such that greater BMI was weakly associated with higher fracture risk. For example, among women HR for major osteoporotic fracture per unit greater BMI was 1.01 (1.00, 1.02) and for men 1.02 (1.01, 1.03) (Table 1). Overall, associations were similar between men and women (Table S2).

**Table 1:** Hazard ratio (HR) and 95% CI for fracture at the sites indicated per unit greater BMI (linear models). HRs are adjusted for age and time since baseline.

	**Adjusted for age and time since baseline**			**Adjusted for age and time since baseline—for those with BMD**			**Adjusted for age, time since baseline and BMD**		
**Outcome fracture**	**Number of cohorts**	***I*** ^**2**^	**HR (95% CI)**	**Number of cohorts**	***I*** ^**2**^	**HR (95% CI)**	**Number of cohorts**	***I*** ^**2**^	**HR (95% CI)**
**Female**									
**Any**	55	73	0.99 (0.98, 0.99)	46	65	0.98 (0.98, 0.99)	46	42	1.01 (1.01, 1.01)
**Hip**	57	57	0.95 (0.94, 0.95)	46	41	0.95 (0.94, 0.96)	46	33	0.99 (0.98, 1.00)^0.060^
**MOF**	52	83	0.98 (0.97, 0.98)	45	77	0.98 (0.97, 0.98)	45	57	1.01 (1.00, 1.02)^0.0018^
**Ost**	51	83	0.98 (0.97, 0.98)	44	73	0.98 (0.97, 0.98)	44	55	1.01 (1.00, 1.01)^0.0051^
**Male**									
**Any**	38	25	0.99 (0.98, 0.99)	32	0	0.99 (0.98, 0.99)	32	0	1.02 (1.01, 1.02)
**Hip**	36	48	0.95 (0.93, 0.96)	29	32	0.95 (0.93, 0.97)	28	0	1.03 (1.01, 1.04)
**MOF**	35	60	0.98 (0.97, 0.98)	30	0	0.98 (0.97, 0.98)	30	1	1.02 (1.01, 1.03)
**Ost**	34	56	0.98 (0.97, 0.99)	30	18	0.98 (0.97, 0.99)	30	0	1.02 (1.01, 1.03)

Except where indicated, *p* < .001.

Abbreviations: MOF, major osteoporotic fracture; Ost, osteoporotic fracture.

### Categorical and non-linear associations between BMI and risk of incident fracture

**Figure 2:**
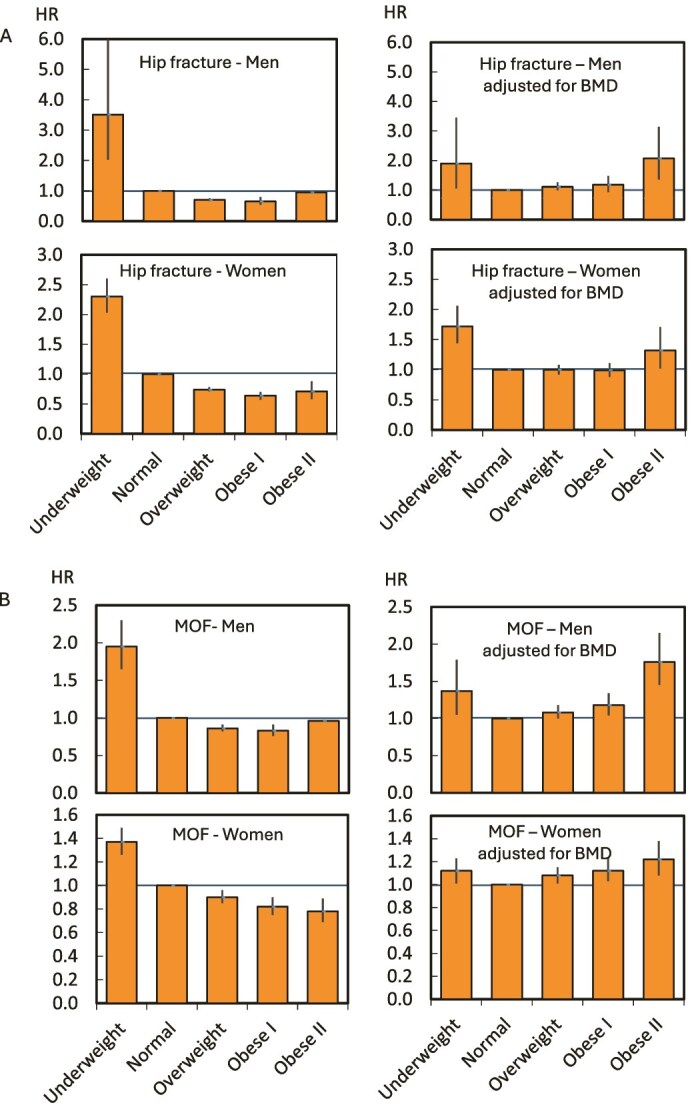
Hazard ratio (HR) (95% CI for fracture according to categories of BMI (referent: normal BMI) for men and women. (A) Hip fracture (HF) and (B) major osteoporotic fracture (MOF). Associations are adjusted for age and follow-up time +/− FN BMD T-score, in subset with FN BMD measure available.

On average, those who were underweight tended to be somewhat older than those in other categories and had lower FN BMD T-score (Table S3). Indeed, there was modest but positive gradient of mean FN BMD T-score going from underweight to normal to overweight to obese categories. Tables 2 and 3 and Figure 2 document the associations between BMI category relative to the normal BMI for men and women, in the subset with BMD measurements, unadjusted and adjusted for FN BMD. As with the initial models, associations were not materially different in the subset with BMD, without BMD adjustment, but the increased risk associated with low BMI and the more modest protective effect of higher BMI category was attenuated by adjustment for FN BMD. Indeed, after BMD adjustment, obesity became a risk factor for fracture in both sexes, with evidence of a greater effect size in men than women (Tables 2 and 3, Table S4). Figure S1 presents the continuous relationships between BMI (HR for fracture for a given BMI vs a BMI of 25 kg/m^2^) and risk of HF in men and women separately, in the subset with BMD, without and then with adjustment for FN BMD.

**Table 2:** Hazard ratio (HR) for fracture by BMI category (reference normal) in women and men, adjusted for age and time since baseline, in subset with FN BMD measure available (but not adjusted for BMD). (BMI categories: underweight BMI < 18.5, normal BMI 18.5-24.9, overweight BMI 25.0-29.9, obese I BMI 30.0-34.9, obese II BMI ≥ 35.0 kg/m^2^). HR in bold are <0.001.

		**1**	**3**	**4**	**5**
		**Underweight vs Normal**	**Overweight vs Normal**	**Obese I vs Normal**	**Obese II vs Normal**
**Outcome fracture**	*N*, cohorts	HR (95% CI)	HR (95% CI)	HR (95% CI)	HR (95% CI)
**Women**					
** Any**	38, 47, 45, 40	**1.31 [1.20**, **1.43]**	**0.94 [0.90**, **0.98]**	**0.88 [0.83, 0.93]**	**0.84 [0.77, 0.91]**
** Hip**	27, 41, 33, 22	**2.35 [2.13**, **2.60]**	**0.75 [0.72, 0.78]**	**0.64 [0.59, 0.69]**	**0.66 [0.55, 0.80]**
** MOF**	33, 45, 42, 36	**1.43 [1.30, 1.59]**	**0.90 [0.85, 0.95]**	**0.82 [0.76, 0.89]**	**0.78 [0.70, 0.88]**
** Ost**	34, 44, 42, 36	**1.38 [1.27, 1.50]**	**0.90 [0.86, 0.94]**	**0.84 [0.79, 0.90]**	**0.77 [0.70, 0.85]**
**Men**					
** Any**	21, 30, 27, 23	**1.77 [1.54**, **2.04]**	**0.87 [0.84**-**0.90]**	**0.88 [0.82, 0.94]**	1.04 [1.04, 1.05]
** Hip**	14, 23, 16, 11	**2.45 [1.90**, **3.17]**	**0.69 [0.65-0.74]**	**0.63 [0.53, 0.74]**	0.91 [0.66, 1.26]
** MOF**	16, 28, 23, 17	**1.87 [1.60, 2.20]**	**0.85 [0.80-0.90]**	**0.80 [0.72, 0.89]**	**0.96 [0.96**, **0.97]**
** Ost**	19, 28, 25, 21	**1.83 [1.58**, **2.12]**	**0.85 [0.82**-**0.89]**	**0.82 [0.76**, **0.89]**	**0.96 [0.96**, **0.97]**
***p* women vs men**					
** Any**		**<0.001**	**0.0067**	>0.30	**<0.001**
** Hip**		>0.30	**0.024**	>0.30	0.088
** MOF**		**0.0040**	0.18	>0.30	**<0.001**
** Ost**		**0.0011**	0.053	>0.30	**<0.001**

Abbreviations: MOF, major osteoporotic fracture; Ost, osteoporotic fracture.

**Table 3:** Hazard ratio (HR) for fracture by BMI category (reference normal) in women and men, adjusted for age and time since baseline, in subset with FN BMD measure available, additionally adjusted for BMD. (BMI categories: underweight BMI < 18.5, normal BMI 18.5-24.9, overweight BMI 25.0-29.9, obese I BMI 30.0-34.9, obese II BMI ≥ 35.0 kg/m^2^). HR in bold are <0.001.

		**1**	**3**	**4**	**5**
		**Underweight vs Normal**	**Overweight vs Normal**	**Obese I vs Normal**	**Obese II vs Normal**
**Outcome fracture**	*N*, cohorts	HR (95% CI)	HR (95% CI)	HR (95% CI)	HR (95% CI)
**Women**					
** Any**	38, 47, 45, 40	**1.09 [1.03**, **1.17]**	**1.08 [1.04**, **1.12]**	**1.13 [1.07**, **1.20]**	**1.19 [1.10**, **1.29]**
** Hip**	27, 41, 33, 22	**1.69 [1.47**, **1.96]**	0.99 [0.93, 1.05]	0.97 [0.89, 1.06]	1.24 [0.97, 1.58]
** MOF**	33, 45, 42, 36	**1.16 [1.08**, **1.25]**	**1.07 [1.01**, **1.13]**	**1.12 [1.04**, **1.21]**	**1.21 [1.09**, **1.35]**
** Ost**	34, 44, 42, 36	**1.13 [1.06**, **1.21]**	**1.06 [1.01**, **1.11]**	**1.13 [1.05**, **1.21]**	**1.17 [1.05**, **1.29]**
**Men**					
** Any**	21, 30, 27, 23	**1.23 [1.00**, **1.51]**	1.03 [0.97, 1.09]	**1.14 [1.05**, **1.24]**	**1.43 [1.24**, **1.65]**
** Hip**	14, 23, 16, 11	1.46 [1.00, 2.13]	1.06 [0.95, 1.18]	1.10 [0.87, 1.38]	**1.70 [1.06**, **2.75]**
** MOF**	16, 28, 23, 17	1.25 [0.98, 1.60]	1.06 [0.98, 1.15]	**1.14 [1.01**, **1.29]**	**1.54 [1.26**, **1.88]**
** Ost**	19, 28, 25, 21	**1.23 [1.00**, **1.52]**	1.03 [0.96, 1.09]	**1.11 [1.02**, **1.22]**	**1.53 [1.27**, **1.83]**
***p* women vs men**					
** Any**		0.27	0.19	>0.30	**0.027**
** Hip**		>0.30	0.29	>0.30	0.25
** MOF**		>0.30	>0.30	>0.30	**0.037**
** Ost**		>0.30	>0.30	>0.30	**0.045**

Abbreviations: MOF, major osteoporotic fracture; Ost, osteoporotic fracture.

### Ethnicity

Table S5 documents the fracture outcome models with continuous BMI as the exposure, by ethnicity (HR and 95%CI fracture risk for each one-unit greater BMI for White, Asian, Black, and Hispanic ethnicities, together with *p* value for a BMI•ethnicity interaction). There was no statistical evidence for a difference in the association between BMI and HF by ethnicity. In contrast to White ethnicity, for Asian and Hispanic ethnicities, higher BMI was associated with a greater risk of any fracture [Asian: HR (95% CI) 1.02 (1.00, 1.04); Hispanic 1.02 (1.00, 1.05)].

### Interaction with age and follow-up time

For all, osteoporotic and MOF fracture outcomes, the HR for fracture per one-unit greater BMI tended to decrease with increasing age. Conversely, for the outcome of HF, there was no obvious relationship with age. (Table S6). There was a modest increase in HR with greater follow-up time for the HF and MOF in the whole cohort after adjusting for age (Table S7).

### Association between BMI and mortality

Compared to normal weight, both underweight [HR (95%CI): 1.60 (1.44, 1.78) in women and 1.71 (1.50, 1.94) in men] and obese I and II categories [e.g., obese II: 1.31 (1.21, 1.43) in women and 1.27 (1.07, 1.51) in men] were associated with greater risk of death during follow-up, in both sexes. Overweight was not associated with increased mortality risk and indeed was protective in women [0.92 (0.88, 0.95)] and men [0.96 (0.92, 1.00)]. Overall associations were similar after adjustment for FN BMD T-score. Table S8A and B summarizes these associations.

## Discussion

### Summary of findings

The present study represents the largest meta-analysis to date on the association between BMI and subsequent fracture risk. It confirms our previous observation in women that low BMI is associated with increased fracture risk, persisting after adjustment for FN BMD.^2^ Furthermore, it confirms in women that overall higher BMI becomes a risk factor for fracture after adjustment for BMD, despite being protective when used unadjusted. In addition to these confirmatory findings, our new analysis documents important novel associations in men: although we observed a similar pattern overall to that in women, the increased risk of fracture associated with obesity after adjustment for BMD was of greater magnitude in men compared with women.

### Comparison with previous studies

This analysis provides an updated comparison with our previous meta-analysis, which examined relationships between BMI and fracture risk only in women, based on a much smaller cohort database with substantially less time at risk available.^2^ There, we observed that low BMI was a risk factor for hip and all osteoporotic fractures; when adjusted for BMD, it remained a risk factor for HF but was weakly protective for osteoporotic fracture. In contrast, high BMI, when adjusted for BMD, was a risk factor for osteoporotic fractures. Our initial 2005 meta-analysis focused on both men and women but with a smaller number of participants and, while the results were broadly comparable, was not able to elucidate any differences by sex.^12^ Similar adverse effect of high BMI on fracture risk, after adjustment for BMD, were observed in the US MrOS cohort.^23^ Compston et al. examined the relationship between BMI and fracture outcomes in the GLOW observing, in 52 939 postmenopausal women, a protective association between greater BMI and risk of hip, clinical spine, and wrist fractures.^24^ In contrast, higher BMI was associated weakly with greater risk of ankle fracture. While this study has sometimes been interpreted by others as demonstrating that obesity is not protective for osteoporotic fracture, there is clearly a question of whether ankle fractures are a consequence of osteoporosis per se, given that the age and sex relationship differs from that with classical osteoporotic fractures.^17^^,^^18^^,^^25–27^ Thus rather than being protective for ankle fractures, obesity appears to be a risk factor for these events. The findings are overall consistent with our current meta-analysis in suggesting that associations between BMI and fracture risk are site specific, with potential further mechanistic implications.

While factors, such as chronic inflammation, type 2 diabetes, and propensity to falls, may contribute to fracture risk in obesity,^5^^,^^28^ other considerations may underpin apparent sex differences observed. For example, in the present analysis, the greater risk of HF in men compared with women at high BMI after consideration of FN BMD may reflect sex dependent distribution of adipose tissue, with the female distribution more classically subcutaneous around the hips than abdomen, compared with the visceral abdominal deposition in men.^29^^,^^30^ Biomechanically, the additional adipose tissue overlying the proximal femur would favor a protective effect for HF in women compared with men. Additionally, or alternatively, a further mechanism might relate to sex-dependent differential hormonal patterns in obesity. Excess adipose tissue is associated with increased estrogen activity, with likely positive effects on bone and muscle.^31^ However, in men, obesity may lead to a concomitant reduction in male sex steroids and thus potentially effective hypogonadism,^32^^,^^33^ with increased fracture risk possibly additionally mediated via impaired muscle health in addition to reduced BMD.^34^

### Strengths and limitations

We undertook the largest meta-analysis to date of mostly population-based cohorts to investigate and quantify associations between BMI and risk of incident fractures. We were able to investigate BMI as a continuous exponential, nonlinear and categorical exposure, and investigate possible interactions by age, sex, ethnicity, and follow-up time. The use of primary data decreases the risk of publication bias and the general consistency of the BMI-fracture association between cohorts provides strong support for the international validity of this risk variable. However, there are limitations which need to be considered in the interpretation of our findings. First, as with nearly all population-based studies, non-response biases may have occurred, which we were unable to document for all cohorts. This is likely to result in a cohort which is healthier than the background population and may thus lead to underestimation of the absolute risk of fracture. In particular, there may have been reduced responses from those at the lowest and highest BMI values. It is therefore likely, if anything, to have had a conservative effect on our risk estimates. Second, it is possible that there were differences in protocols and, for example, execution of height and weight measures between cohorts. This is unavoidable in such a study, and importantly, exposure-outcome analyses were undertaken within each cohort and then the beta coefficients merged through random effects meta-analysis. Third, we were not able to elucidate whether the association between BMI and incident fracture was causal. However, this was not the purpose of the study and for the purpose of risk assessment, a causal relationship is not required. Fourth, BMI is affected by both fat and lean mass and does not account for distribution of fat between subcutaneous and visceral compartments. This is irrelevant in terms risk assessment, but of course limits mechanistic influence. Finally, we were not able to account for levels of physical activity, which might be related to both BMI and fracture risk. Although this might be mechanistically interesting, it would not reflect the way in which BMI is considered in the FRAX algorithm.

### Clinical and risk assessment implications

The role of BMI in the current FRAX model was informed by an earlier meta-analysis by De Laet et al.,^12^ which was broadly consistent with the Johansson et al., 2014 study,^2^ the conclusion at that time being that the new findings did not necessitate modification of the FRAX engine itself. Here we present novel associations relating to men as well as women, with interactions by sex, follow-up time, ethnicity, and age, which will directly inform the revised FRAX engine, as a continuous exposure, in the second version of this globally established tool. Importantly our current findings also confirm the non-linear association between BMI and fracture. The variably modifying effects of age, ethnicity, and follow-up time, together with differences by sex, support the accommodation of such nuances in the FRAX risk engine, but conversely mitigate against using BMI thresholds in risk assessment, since these would not necessarily convey consistent risk information across these parameters or fracture outcomes. The associations between increased risk of death during follow-up and underweight, and to a lesser extent obesity, are consistent with a large recent analyses,^35^ and will also be important to consider in the derivation of the revised FRAX tool.

While there are likely to be causal implications, underpinned by our understanding of biology and biomechanics, for the purposes of risk assessment, the principal requirement is quantification of the exposure-outcome association and of interactions between this relationship and with other potential input variables. Notwithstanding, our findings directly inform clinical practice, indicating that greater BMI is not necessarily protective for all fracture types. While this may play into strategies to tackle obesity, there is evidence that weight loss usually leads to concomitant loss of muscle and bone and so approaches addressing obesity in the context of fracture risk must be carefully thought out.^36^ Adequate calcium and protein nutrition, together with a mix of weight-bearing and resistance exercise training, alongside dietary interventions to reduce calorie intake are likely to be important,^37^ given the clear demonstration of increasing risk of HF with lower BMI, even after adjustment for BMD.

## Conclusion

In the largest meta-analysis to date of prospective cohorts worldwide, we have confirmed inverse associations between fracture risk and BMI, with the apparent protective effect of higher BMI reversed after adjustment for FN BMD. The magnitude of this effect appeared greater in men than women. The increased accuracy of the elucidated effect sizes, and their specificity by age, sex, follow-up time, and ethnicity will inform improved risk assessment in the second iteration of the FRAX tool.

## Human and animal rights

This review does not contain any original studies with human participants or animals performed by any of the authors.

## Ethics

All individual cohorts with candidate risk factors available have been approved by their local ethics committees and informed consent has been obtained from all study participants. General ethics approval for the use of these cohorts is also given by the University of Sheffield. Participant data are stored in coded, de-identified form. Only summary statistics and aggregate data are published, not allowing for identification of individual study participants.

## Supplementary Material

BMI_FRAX_Online_Supplementary_material_zjaf091

## Data Availability

Data availability is cohort specific and individual cohort Principal Investigators should be contacted for enquiries about data access.
