# Supplementary material for: Body mass index and subsequent fracture risk: a meta-analysis to update FRAX
Source: J Bone Miner Res. 2025 Aug 8;40(10):1144–55. doi: 10.1093/jbmr/zjaf091 (PMC12487781; doi:10.1093/jbmr/zjaf091)
Supplement: BMI_FRAX_Online_Supplementary_material_zjaf091 [file bmi_frax_online_supplementary_material_zjaf091.docx]

**Body mass index and subsequent fracture risk: A meta-analysis to update FRAX^®^: Online Supplementary Material**

**Appendix Table 1:** Characteristics of the cohorts studied.

|  |  |  |  | Age | | |  | BMI | | | Incident fractures | | | | |
| --- | --- | --- | --- | --- | --- | --- | --- | --- | --- | --- | --- | --- | --- | --- | --- |
|  | n | BMD n | Person years | Mean | Min | Max | % women | Mean | Min | Max | Hip | Any | MOF | MOF  wo Hip | Ost |
| AGES | 5640 | 4778 | 45247 | 76.9 | 66 | 96 | 57.5 | 27.0 | 13.6 | 49.7 | 525 | 1606 | 1123 | 763 | 1384 |
| AHS | 2603 | 2588 | 10073 | 65.1 | 47 | 95 | 69.5 | 23.0 | 14.2 | 42.1 | 25 | 322 | 236 | 218 | 236 |
| APOSS | 5105 | 4979 | 37311 | 48.5 | 44.2 | 56.3 | 100 | 25.5 | 15.2 | 56.8 | 4 | 336 | 143 | 142 | 177 |
| AUSTRIOS_B | 1932 | 0 | 2247 | 83.9 | 68 | 103 | 83.9 | 25.6 | 13.8 | 46.1 | 75 | 171 | - | - | - |
| BEH | 2298 | 2291 | 10192 | 69.3 | 60 | 96 | 51.4 | 27.5 | 12.2 | 53.3 | 40 | 98 | 46 | 5 | 76 |
| BERN | 17610 | 17355 | 140261 | 58.9 | 20 | 94.3 | 84.0 | 24.9 | 11.6 | 52.1 | 252 | 4374 | 2503 | 2339 | 3355 |
| CAMOS | 9146 | 8272 | 119146 | 62.0 | 25 | 103 | 69.4 | 27.0 | 13.0 | 74.9 | 334 | 2390 | 1160 | 910 | 1717 |
| DOES | 2077 | 2048 | 19248 | 70.1 | 47 | 94 | 60.9 | 25.6 | 14.3 | 47.3 | 95 | 478 | 297 | 236 | 402 |
| DO_HEALTH | 2147 | 1444 | 5932 | 75.0 | 70 | 95 | 61.7 | 26.3 | 15.2 | 48.3 | 10 | 266 | 118 | 111 | 191 |
| ECOSAP | 5128 | 0 | 16797 | 72.3 | 65 | 100 | 100 | 29.2 | 15.4 | 50.6 | 52 | 310 | 187 | 135 | 258 |
| EPIC_NORFOLK | 25576 | 0 | 493213 | 59.2 | 39 | 79 | 54.7 | 26.4 | 15.2 | 58.7 | 1353 | 3027 | 2335 | 1198 | - |
| EPIDOS | 7591 | 7558 | 21188 | 80.5 | 70.1 | 100.0 | 100 | 25.4 | 13.1 | 51.8 | 226 | 1026 | 568 | 376 | 837 |
| EPIFROS | 284 | 12 | 2826 | 61.6 | 40 | 96 | 54.6 | 27.2 | 16.2 | 54.2 | 3 | 27 | 16 | 13 | 20 |
| EVOS_EPOS | 13490 | 4746 | 41495 | 63.9 | 40.6 | 91.4 | 51.7 | 27.1 | 14.2 | 52.5 | 50 | 543 | 292 | 245 | 543 |
| FORMEN | 1886 | 1882 | 16265 | 72.5 | 65 | 93 | 0 | 22.9 | 14.7 | 33.6 | 10 | 90 | 58 | 49 | 90 |
| FRAM_OFF | 3465 | 2938 | 47025 | 61.4 | 33 | 90 | 53.8 | 28.1 | 15.8 | 58.2 | 89 | 680 | 270 | 206 | 523 |
| FRAM_ORIG | 1045 | 896 | 9179 | 79.2 | 72 | 100 | 64.0 | 26.7 | 12.7 | 55.6 | 106 | 250 | 159 | 66 | 227 |
| FRIDEX | 815 | 815 | 8077 | 56.8 | 40 | 84 | 100 | 28.4 | 17.8 | 49.4 | 15 | 112 | 41 | 28 | 56 |
| FROCAT | 1936 | 237 | 19235 | 69.2 | 32 | 111 | 55.5 | 27.7 | 14.8 | 52.0 | 32 | 226 | 158 | 133 | 181 |
| GERICO | 763 | 750 | 2763 | 67.9 | 64.6 | 72.2 | 79.4 | 25.4 | 16.2 | 45.3 | 2 | 71 | 26 | 24 | 51 |
| GLOW | 51887 | 0 | 207881 | 68.1 | 55 | 108 | 100 | 26.9 | 7.5 | 78.7 | 454 | 5456 | 2720 | 2342 | 4089 |
| GOS | 1861 | 1803 | 12811 | 63.0 | 35 | 95.3 | 100 | 26.8 | 14.7 | 66.6 | 32 | 174 | 115 | 89 | 150 |
| GOTHENBURG_I | 1344 | 0 | 8518 | 85.3 | 70.1 | 96.0 | 53.1 | 25.2 | 12.8 | 46.8 | 232 | 339 | 283 | 88 | 317 |
| GOTHENBURG_II | 11338 | 0 | 149379 | 59.0 | 21 | 84 | 100 | 24.4 | 14 | 49 | 257 | 1187 | 735 | 642 | 851 |
| HAI | 3617 | 3535 | 9510 | 70.5 | 69.2 | 72.0 | 50.2 | 26.6 | 16.0 | 54.5 | 10 | 126 | 78 | 68 | 114 |
| HCS | 632 | 631 | 5595 | 64.9 | 59.1 | 70.9 | 50.3 | 26.8 | 17 | 48.3 | 3 | 67 | 35 | 33 | 51 |
| HEALTH_ABC | 3075 | 3043 | 36485 | 73.6 | 68 | 80 | 51.5 | 27.4 | 14.6 | 52.0 | 235 | 701 | 521 | 352 | 597 |
| JPOS | 1944 | 1933 | 25812 | 57.5 | 40 | 82 | 100 | 23.8 | 15.4 | 43.4 | 29 | 265 | 99 | - | - |
| LASA | 1453 | 518 | 7500 | 75.6 | 64.8 | 88.7 | 51.6 | 26.9 | 14.4 | 49.3 | 39 | 131 | - | - | 95 |
| Maccabi | 407816 | 28620 | 3880004 | 57.5 | 34 | 91 | 52.5 | 28.3 | 5.1 | 64.0 | 8271 | 38825 | 37132 | 30493 | 38537 |
| Manitoba | 92281 | 92106 | 833466 | 63.4 | 20 | 104 | 89.1 | 27.3 | 10.3 | 72.0 | 3085 | 13506 | 9578 | 7187 | 12655 |
| MINOS | 672 | 664 | 6096 | 65.2 | 50 | 86 | 0 | 28.0 | 18.3 | 44.7 | 3 | 62 | 24 | 21 | 55 |
| MIYAMA | 400 | 400 | 3703 | 59.1 | 40 | 79 | 50 | 22.1 | 14.8 | 31.6 | 7 | 61 | 35 | 30 | 47 |
| MROS_HONGKONG | 2000 | 2000 | 19744 | 72.4 | 65 | 92 | 0 | 23.4 | 13.1 | 36.3 | 63 | 231 | 148 | 93 | 201 |
| MROS_USA | 5992 | 5991 | 74985 | 73.7 | 64 | 100 | 0 | 27.4 | 17.2 | 50.7 | 330 | 1394 | 814 | 490 | 1082 |
| MSOS_HONGKONG | 2000 | 2000 | 17528 | 72.6 | 65 | 98 | 100 | 23.9 | 12.7 | 40.4 | 69 | 338 | 247 | 189 | 298 |
| NHEFS | 12207 | 0 | 121645 | 49.4 | 25 | 74 | 59.6 | 25.7 | 12.4 | 72.3 | 113 | - | - | - | - |
| OFELY | 867 | 861 | 15136 | 58.8 | 40 | 89 | 100 | 23.8 | 16.6 | 38.4 | 40 | 245 | 180 | 159 | 207 |
| OPRA | 1024 | 947 | 11996 | 75.2 | 75.0 | 76.0 | 100 | 26.3 | 14.9 | 49.8 | 193 | 517 | 447 | - | 467 |
| OPUS | 2008 | 1979 | 12322 | 62.1 | 20.2 | 80.6 | 100 | 26.5 | 16.2 | 46.1 | 14 | 241 | 117 | 106 | 153 |
| OSTEOLAUS | 1475 | 1457 | 6726 | 64.5 | 50.2 | 81.5 | 100 | 25.9 | 15.3 | 39.9 | 8 | 307 | 226 | 221 | 245 |
| OSTPRE | 11195 | 2760 | 109415 | 57.3 | 52.4 | 62.7 | 100 | 27.1 | 14.3 | 61.0 | 75 | 1846 | 913 | 845 | 1254 |
| PERF | 5412 | 2564 | 36195 | 63.9 | 43.6 | 80.8 | 100 | 25.5 | 14.1 | 47.0 | 56 | 784 | 511 | 461 | 516 |
| REFORM | 971 | 0 | 1435 | 77.8 | 65 | 99 | 60.4 | 27.6 | 15.0 | 62.2 | 3 | 27 | 11 | 8 | 15 |
| ROCHESTER | 1001 | 993 | 7686 | 56.8 | 21.0 | 94.2 | 65.2 | 26.1 | 14.0 | 51.7 | 37 | 326 | 243 | 229 | 283 |
| ROTTERDAM | 13060 | 10981 | 146503 | 65.0 | 45.5 | 99.1 | 57.6 | 26.9 | 12.6 | 56.9 | 710 | 2999 | 2081 | 1590 | 2599 |
| SAOL_IPR_EPIP | 925 | 923 | 11228 | 55.9 | 40 | 89 | 77.4 | 27.2 | 15.6 | 49.9 | 12 | 105 | 41 | 18 | - |
| SARCOPHAGE | 228 | 217 | 440 | 75.9 | 68.2 | 93.4 | 57.0 | 26.8 | 15.6 | 44.5 | 1 | 13 | 5 | 4 | 8 |
| SCOOP | 12483 | 2818 | 59368 | 75.6 | 70 | 86 | 100 | 26.7 | 12.8 | 66.4 | 382 | 1952 | 1298 | 986 | 1646 |
| SEMOF | 7133 | 920 | 20631 | 75.2 | 70 | 91.3 | 100 | 25.9 | 12.6 | 57.5 | 80 | 683 | 464 | 384 | 596 |
| SHEFFIELD | 2152 | 2152 | 7373 | 80.0 | 74.3 | 100.9 | 100 | 26.6 | 14.8 | 47.1 | 67 | 288 | 190 | 135 | 233 |
| SINGAPORE | 52042 | 0 | 462436 | 61.6 | 48 | 84 | 57.4 | 23.2 | 10.0 | 45.4 | 1091 | - | - | - | - |
| SOF | 9485 | 7605 | 133919 | 71.6 | 65 | 89 | 100 | 26.4 | 16.8 | 43.3 | 1378 | 4267 | 2752 | 1813 | 3396 |
| SOS | 16626 | 4080 | 62119 | 74.2 | 60.8 | 92.5 | 100 | 26.8 | 13.1 | 138.8 | 260 | 1383 | 993 | 702 | 1325 |
| STOP_IT | 424 | 424 | 1840 | 71.1 | 65 | 87.1 | 55.0 | 26.8 | 16.6 | 46.6 | 2 | 50 | 24 | 22 | 32 |
| STRAMBO | 823 | 804 | 7582 | 72.1 | 51 | 88.4 | 0 | 27.7 | 18.5 | 42.7 | 17 | 117 | 42 | 26 | 86 |
| TASOAC | 1098 | 1093 | 10955 | 63.0 | 50.8 | 80.9 | 48.9 | 27.9 | 17.6 | 52.9 | 5 | 146 | 49 | 46 | 88 |
| THIN | 180120 | 0 | 882650 | 60.4 | 50 | 105 | 100 | 26.0 | 10.5 | 99.4 | 1954 | 11959 | - | - | 8344 |
| WHI | 80949 | 6109 | 1109973 | 64.3 | 49 | 79 | 100 | 28.5 | 11.9 | 69.6 | 2366 | 6802 | 4649 | 2513 | 5329 |
| HUNT | 49894 | 11556 | 618982 | 53.2 | 20 | 100.8 | 54.6 | 27.2 | 12.1 | 55.9 | 1646 | 10153 | 4694 | 3579 | 7063 |
| MRSWE | 3012 | 2819 | 34202 | 74.9 | 69 | 81 | 0 | 26.4 | 13.3 | 44.6 | 340 | 971 | 730 | 483 | 876 |
| UKBIO | 499431 | 19415 | 5732600 | 56.5 | 37 | 73 | 54.5 | 27.4 | 12.1 | 74.7 | 3878 | 24943 | 11980 | 8278 | 19868 |
| SUPERB | 3028 | 3015 | 23448 | 77.8 | 74.7 | 81.0 | 100 | 26.3 | 16.2 | 47.9 | 238 | 1083 | 808 | 654 | 1043 |
| **Total** | **1 667 922** | **293 325** | **16 015 542** | **59.8** | **20** | **111** | **67.6** | **27.2** | **5.1** | **138.8** | **31 383** | **151 473** | **95 748** | **72 576** | **125 135** |

MOF, major osteoporotic fracture; AGES, Age, Gene/Environment Susceptibility‐Reykjavik Study; AHS, Adult Health Study; APOSS, Aberdeen Prospective Osteoporosis Screening Study; BEH, Bushehr Elderly Health; CaMos, Canadian Multicentre Osteoporosis Study; DOES, Dubbo Osteoporosis Epidemiology Study; DO-HEALTH, VitaminD3-Omega3-Home Exercise-Healthy Aging and Longevity Trial; ECOSAP, Ecografía Osea en Atención Primaria; EPIC-Norfolk, European Prospective Investigation of Cancer-Norfolk; EPIDOS, Epidémiologie de l'Ostéoporose; EPIFROS, EPIdemiology and Fracture Risk factors for Osteoporosis in Spain; EVOS/EPOS, European Vertebral Osteoporosis Study/European Prospective Osteoporosis Study; FORMEN, Fujiwara-kyo Osteoporosis Risk in Men; FRIDEX, Fracture RIsk factors and bone DEnsitometry type central dual X-ray; FROCAT, Fracture Risk factors for Osteoporosis in CATalonia; GERICO, Geneva Retirees Cohort; GLOW, Global Longitudinal Study of Osteoporosis in Women; GOS, Geelong Osteoporosis Study; HAI, Healthy Ageing Initiative; HCS, Hertfordshire Cohort Study; Health ABC, Health, Aging and Body Composition; HUNT, The Trøndelag Health Study; JPOS, Japanese Population-based Osteoporosis Study; LASA, Longitudinal Aging Study Amsterdam; MINOS, Montceau les MINes OSteoporosis; MrOS, Osteoporotic Fractures in Men; MsOS, Osteoporotic Fractures in Women; NHEFS, National Health and Nutrition Examination Survey (NHANES) I Epidemiologic Follow-up Study; OFELY, Os des Femmes de Lyon; OPRA, Osteoporosis Prospective Risk Assessment; OPUS, Osteoporosis and Ultrasound Study; OSTPRE, Kuopio OSTeoporosis risk factor and PREvention study; PERF, Prospective Epidemiologic Risk Factor; REFORM, REducing Falls with ORthoses and a Multifaceted podiatry intervention; SAOL-IPR-EPIPorto, Santo António dos Olivais, Instituto Português de Reumatologia and EPIPorto; SarcoPhAge, Sarcopenia and Physical Impairment with advancing Age; SCHS, Singapore Chinese Health Study; SCOOP, screening for prevention of fractures in older women; SEMOF, Swiss Evaluation of the Methods of Measurement of Osteoporotic Fracture risk; SOF, Study of Osteoporotic Fractures; SOS, SALT Osteoporosis Study; STRAMBO, Structure of the Aging Men’s Bone; SUPERB, Sahlgrenska University hospital Prospective Evaluation of Risk of Bone fractures; TASOAC, Tasmanian Older Adult Cohort; THIN, The Health Improvement Network; WHI, Women’s Health Initiative.

**Appendix Table 2:** Hazard ratio (HR) and 95% confidence interval (CI) for fracture per unit greater BMI in men and women in those cohorts that contributed both men and women (linear models). HRs are adjusted for age and time since baseline.

| **Adjusted for BMD** | **Outcome**  **fracture** | **Men** |  |  | **Women** |  | **Number of cohorts** | **p-interaction** |
| --- | --- | --- | --- | --- | --- | --- | --- | --- |
|  |  | *HR (95%CI)/unit* |  |  | *HR (95%CI)/unit* |  |  |  |
| No | Any | 0.99 (0.98, 0.99) |  |  | 0.99 (0.98, 0.99) |  | 33 | 0.54 |
|  | Hip | 0.94 (0.93, 0.96) |  |  | 0.95 (0.94, 0.96) |  | 31 | 0.49 |
|  | MOF | 0.98 (0.96, 0.99) |  |  | 0.98 (0.97, 0.99) |  | 30 | 0.16 |
|  | Ost | 0.98 (0.97, 0.99) |  |  | 0.98 (0.98, 0.98) |  | 29 | 0.74 |
|  |  |  |  |  |  |  |  |  |
| Yes | Any | 1.01 (1.01, 1.02) |  |  | 1.01 (1.01, 1.01) |  | 27 | 0.18 |
|  | Hip | 1.00 (0.98, 1.02) |  |  | 0.99 (0.98, 1.00) |  | 23 | 0.35 |
|  | MOF | 1.01 (1.00, 1.01) |  |  | 1.01 (1.00, 1.01) |  | 25 | 0.91 |
|  | Ost | 1.01 (1.01, 1.02) |  |  | 1.01 (1.00, 1.01) |  | 25 | 0.18 |

MOF = major osteoporotic fracture; Ost = osteoporotic fracture

**Appendix Table 3:** Baseline characteristics by body mass index (BMI, kg/m^2^) category, mean (SD).

|  | **Underweight**  **(<18.5)** | **Normal**  **(18.5-24.9)** | **Overweight**  **(25.0-29.9)** | **Obese I**  **(30.0-34.9)** | **Obese II (≥35.0)** |
| --- | --- | --- | --- | --- | --- |
| *Men* | *N=3605* | *N=97 363* | *N=152 320* | *N=56 465* | *N=15 470* |
| Age (years) | 63.5 (11.1) | 59.0 (11.2) | 58.4 (10.2) | 58.0 (9.6) | 57.1 (9.0) |
| BMI (kg/m^2^) | 17.2 (1.1) | 22.9 (1.6) | 27.3 (1.40) | 31.9 (1.4) | 38.3 (3.5) |
| Femoral neck BMD (T-score) | -1.9 (1.1)  N=615 | -1.1 (1.0)  N=15 426 | -0.6 (1.0)  N=19 581 | -0.3 (1.1)  N=5934 | 0.0 (1.1)  N=1235 |
|  |  |  |  |  |  |
| *Women* | *N=16 823* | *N=339 605* | *N=283 448* | *N=119 465* | *N=57 580* |
| Age (years) | 63.6 (12.9) | 60.4 (11.5) | 61.8 (10.8) | 61.6 (10.3) | 59.9 (9.7) |
| BMI (kg/m^2^) | 17.2 (1.2) | 22.5 (1.7) | 27.2 (1.4) | 32.0 (1.4) | 39.3 (4.5) |
| Femoral neck BMD (T-score) | -2.1 (1.1)  N=3197 | -1.6 (1.0)  N=44 435 | -1.4 (1.0)  N=35 689 | -1.0 (1.1)  N=13 793 | -0.6 (1.1)  N=5177 |

Note: Maccabi, Manitoba and Epic Norfolk not included since only remote access was possible

**Appendix Table 4:** Hazard ratio for fracture by BMI category (reference normal) in in women and men, adjusted for time since baseline, in whole dataset. (BMI Categories: Underweight BMI<18.5, Normal BMI 18.5-24.9, Overweight BMI 25.0-29.9, Obese I BMI 30.0-34.9, Obese II BMI ≥ 35.0 kg/m^2^).

|  |  | **Women** | | | **Men** | | | **P**  **women vs men** |
| --- | --- | --- | --- | --- | --- | --- | --- | --- |
|  | *Outcome* | *Number*  *of cohorts* | *HR (95CI)* | *p-value* | *Number*  *of cohorts* | *HR (95CI)* | *p-value* |  |
| 1: Underweight vs Normal | ANY FX | 46 | **1.32[1.24-1.41]** | **<0.001** | 24 | **1.80[1.59-2.04]** | **<0.001** | **<0.001** |
| 3: Overweight vs Normal | ANY FX | 55 | **0.94[0.91-0.96]** | **<0.001** | 35 | **0.86[0.82-0.90]** | **<0.001** | **0.0025** |
| 4: Obese I vs Normal | ANY FX | 54 | **0.90[0.86-0.94]** | **<0.001** | 33 | **0.86[0.81-0.92]** | **<0.001** | 0.24 |
| 5: Obese II vs Normal | ANY FX | 50 | **0.84[0.80-0.88]** | **<0.001** | 26 | 0.97[0.89-1.05] | >0.30 | **0.0044** |
| 1: Underweight vs Normal | HIP FX | 38 | **2.11[1.88-2.38]** | **<0.001** | 21 | **2.58[1.95-3.40]** | **<0.001** | 0.19 |
| 3: Overweight vs Normal | HIP FX | 51 | **0.74[0.72-0.76]** | **<0.001** | 29 | **0.71[0.64-0.79]** | **<0.001** | >0.30 |
| 4: Obese I vs Normal | HIP FX | 44 | **0.63[0.59-0.66]** | **<0.001** | 23 | **0.61[0.54-0.69]** | **<0.001** | >0.30 |
| 5: Obese II vs Normal | HIP FX | 33 | **0.59[0.53-0.66]** | **<0.001** | 14 | 0.81[0.62-1.06] | 0.12 | **0.031** |
| 1: Underweight vs Normal | MOF | 39 | **1.46[1.35-1.58]** | **<0.001** | 21 | **2.08[1.65-2.62]** | **<0.001** | **0.0046** |
| 3: Overweight vs Normal | MOF | 51 | **0.90[0.86-0.94]** | **<0.001** | 32 | **0.83[0.76-0.90]** | **<0.001** | 0.11 |
| 4: Obese I vs Normal | MOF | 49 | **0.82[0.77-0.88]** | **<0.001** | 28 | **0.78[0.71-0.87]** | **<0.001** | >0.30 |
| 5: Obese II vs Normal | MOF | 44 | **0.77[0.71-0.84]** | **<0.001** | 22 | **0.89[0.80-1.00]** | **0.048** | **0.034** |
| 1: Underweight vs Normal | OST FX | 41 | **1.38[1.31-1.47]** | **<0.001** | 22 | **1.89[1.60-2.22]** | **<0.001** | **<0.001** |
| 3: Overweight vs Normal | OST FX | 50 | **0.90[0.87-0.93]** | **<0.001** | 31 | **0.82[0.77-0.89]** | **<0.001** | **0.011** |
| 4: Obese I vs Normal | OST FX | 49 | **0.85[0.80-0.89]** | **<0.001** | 29 | **0.79[0.73-0.85]** | **<0.001** | 0.15 |
| 5: Obese II vs Normal | OST FX | 44 | **0.78[0.73-0.83]** | **<0.001** | 22 | 0.91[0.82-1.02] | 0.10 | **0.014** |

**Appendix Table 5:** Hazard ratio (HR) and 95% confidence interval (CI) for fracture at the sites indicated per unit greater BMI in men and women according to ethnicity. HRs are adjusted for age and time since baseline.

| **Outcome fracture** | **Number**  **of cohorts** | **HR** | **95% CI** | **HR** | **95% CI** | **p-value interaction** |
| --- | --- | --- | --- | --- | --- | --- |
| *Asian vs White* | | *White; n=638,922* | | *Asian; n=15,976* | |  |
| Any | 5 | 0.99 | 0.98-1.00 | 1.02 | 1.00-1.04 | **0.0021** |
| Hip | 5 | 0.95 | 0.94-0.97 | 0.95 | 0.90-1.01 | 0.98 |
| MOF | 5 | 0.98 | 0.97-0.99 | 1.01 | 0.98-1.03 | **0.041** |
| *Black vs White* | | *White; n=651,130* | | *Black; n=21,244* | |  |
| Any | 6 | 0.99 | 0.98-1.00 | 1.00 | 0.97-1.02 | 0.68 |
| Hip | 7 | 0.95 | 0.94-0.96 | 0.92 | 0.86-0.98 | 0.31 |
| MOF | 6 | 0.98 | 0.97-0.99 | 0.98 | 0.94-1.01 | 0.91 |
| *Hispanic vs White* | | *White; n=70,248* | | *Hispanic; n=3543* | |  |
| Any | 2 | 0.99 | 0.98-1.01 | 1.02 | 1.00-1.05 | **0.022** |
| Hip | 2 | 0.97 | 0.96-0.98 | 0.97 | 0.91-1.04 | 0.94 |
| MOF | 2 | 0.99 | 0.98-1.00 | 1.05 | 0.95-1.17 | 0.25 |
| *White vs BAME* | | *White; n=662,079* | | *BAME; n=50,078* | |  |
| Any | 8 | 0.99 | 0.98-0.99 | 0.99 | 0.98-0.99 | 0.38 |
| Hip | 8 | 0.95 | 0.94-0.96 | 0.93 | 0.89-0.97 | 0.34 |
| MOF | 7 | 0.98 | 0.97-0.99 | 0.97 | 0.95-0.99 | 0.53 |

**Appendix Table 6**: Hazard ratio (HR) and 95% confidence interval (CI) for fracture at the sites indicated per unit greater BMI in men and women combined, by age. HRs are adjusted for time since baseline and sex. P values refer to the significance of the interaction term with age.

|  | **Any (n=61)** | **Hip (n=59)** | **MOF (n=57)** | **Osteoporotic (n=56)** |
| --- | --- | --- | --- | --- |
| **Age (years)** | *HR/unit* | *HR/unit* | *HR/unit* | *HR/unit* |
| 40 | 1.020 (1.012, 1.028) | 0.939 (0.916, 0.963) | 1.012 (1.001, 1.023) | 1.011 (1.003, 1.020) |
| 45 | 1.015 (1.009, 1.021) | 0.941 (0.921, 0.962) | 1.007 (0.998, 1.016) | 1.007 (1.000, 1.014) |
| 50 | 1.010 (1.005, 1.015) | 0.943 (0.926, 0.961) | 1.002 (0.995, 1.010) | 1.002 (0.997, 1.008) |
| 55 | 1.004 (1.001, 1.008) | 0.945 (0.930, 0.960) | 0.998 (0.992, 1.004) | 0.998 (0.994, 1.002) |
| 60 | 1.000 (0.997, 1.002) | 0.947 (0.935, 0.959) | 0.993 (0.989, 0.997) | 0.993 (0.990, 0.996) |
| 65 | 0.994 (0.992, 0.996) | 0.948 (0.939, 0.958) | 0.989 (0.986, 0.991) | 0.988 (0.987, 0.990) |
| 70 | 0.989 (0.987, 0.991) | 0.950 (0.944, 0.957) | 0.984 (0.982, 0.986) | 0.984 (0.982, 0.986) |
| 75 | 0.984 (0.981, 0.987) | 0.952 (0.948, 0.957) | 0.979 (0.977, 0.982) | 0.979 (0.976, 0.983) |
| 80 | 0.979 (0.974, 0.983) | 0.954 (0.950, 0.958) | 0.975 (0.971, 0.979) | 0.975 (0.970, 0.980) |
| 85 | 0.974 (0.968, 0.979) | 0.956 (0.950, 0.961) | 0.970 (0.965, 0.976) | 0.971 (0.964, 0.977) |
| 90 | 0.969 (0.962, 0.975) | 0.958 (0.950, 0.966) | 0.966 (0.959, 0.973) | 0.966 (0.958, 0.974) |
| P value | <0.001 | 0.23 | <0.001 | <0.001 |

**Appendix Table 7:** Hazard ratio (HR) and 95% confidence interval (CI) for hip and major osteoporotic fractures per unit greater BMI in men and women combined, at increasing years of follow-up time. HRs are adjusted for age and sex. P values refer to the significance of the interaction term with time since baseline.

|  | **Site of outcome fracture** | | | | | | | | | | | |
| --- | --- | --- | --- | --- | --- | --- | --- | --- | --- | --- | --- | --- |
|  | **Any (n=61)** | |  | **Hip (n=59)** | |  | **MOF (n=57)** | |  | **Osteoporotic (n=56)** | | |
| **Time (years)** | *HR/unit* | *95% CI* |  | *HR/unit* | *95% CI* |  | *HR/unit* | *95% CI* |  | *HR/unit* | *95% CI* |  |
| 0 | 0.986 | 0.981-0.990 |  | 0.932 | 0.924-0.939 |  | 0.977 | 0.971-0.984 |  | 0.978 | 0.972-0.984 |  |
| 1 | 0.986 | 0.981-0.990 |  | 0.934 | 0.927-0.941 |  | 0.977 | 0.971-0.984 |  | 0.978 | 0.972-0.984 |  |
| 2 | 0.986 | 0.982-0.989 |  | 0.936 | 0.930-0.942 |  | 0.977 | 0.971-0.984 |  | 0.978 | 0.972-0.984 |  |
| 3 | 0.985 | 0.982-0.989 |  | 0.938 | 0.934-0.943 |  | 0.977 | 0.971-0.984 |  | 0.978 | 0.973-0.984 |  |
| 4 | 0.985 | 0.982-0.989 |  | 0.941 | 0.936-0.945 |  | 0.977 | 0.971-0.984 |  | 0.978 | 0.973-0.984 |  |
| 5 | 0.985 | 0.982-0.988 |  | 0.943 | 0.939-0.947 |  | 0.977 | 0.971-0.984 |  | 0.978 | 0.973-0.984 |  |
| 6 | 0.985 | 0.982-0.988 |  | 0.945 | 0.942-0.949 |  | 0.978 | 0.971-0.984 |  | 0.978 | 0.973-0.983 |  |
| 7 | 0.985 | 0.983-0.988 |  | 0.948 | 0.944-0.952 |  | 0.978 | 0.971-0.984 |  | 0.978 | 0.974-0.983 |  |
| 8 | 0.985 | 0.983-0.988 |  | 0.950 | 0.945-0.955 |  | 0.978 | 0.971-0.984 |  | 0.979 | 0.974-0.983 |  |
| 9 | 0.985 | 0.983-0.988 |  | 0.952 | 0.947-0.958 |  | 0.978 | 0.971-0.984 |  | 0.979 | 0.974-0.983 |  |
| 10 | 0.985 | 0.983-0.987 |  | 0.955 | 0.948-0.961 |  | 0.978 | 0.971-0.984 |  | 0.979 | 0.974-0.983 |  |
| P value | 0.80 |  |  | <0.001 |  |  | 0.0031 |  |  | 0.65 |  |  |

**Appendix Table 8a:** Hazard ratio (HR) and 95% confidence interval (CI) for mortality during follow-up by BMI category (adjusted for age, time since baseline), in the participants for whom BMD is available. Referent: normal BMI category.

|  |  |  | **Women** |  |  | **Men** |  |
| --- | --- | --- | --- | --- | --- | --- | --- |
|  | *Outcome* | *Number*  *of cohorts* | *HR (95CI)* | *p-value* | *Number*  *of cohorts* | *HR (95CI)* | *p-value* |
| 1: Underweight vs Normal | Death | 31 | 1.60 [1.44-1.78] | <0.001 | 21 | 1.71 [1.50-1.94] | <0.001 |
| 3: Overweight vs Normal | Death | 35 | 0.92 [0.88-0.95] | <0.001 | 27 | 0.96 [0.92-1.00] | 0.034 |
| 4: Obese I vs Normal | Death | 31 | 1.08 [1.03-1.14] | 0.0018 | 24 | 1.07 [1.00-1.16] | 0.061 |
| 5: Obese II vs Normal | Death | 28 | 1.31 [1.21-1.43] | <0.001 | 20 | 1.27 [1.07-1.51] | 0.0065 |

**Appendix Table 8b:** Hazard ratio (HR) and 95% confidence interval (CI) for mortality during follow-up by BMI category (adjusted for age, time since baseline and femoral neck BMD T-score), in the participants for whom BMD is available. Referent: normal BMI category.

|  |  |  | **Women** |  |  | **Men** |  |
| --- | --- | --- | --- | --- | --- | --- | --- |
|  | *Outcome* | *Number*  *of cohorts* | *HR (95CI)* | *p-value* | *Number*  *of cohorts* | *HR (95CI)* | *p-value* |
| 1: Underweight vs Normal | Death | 31 | 1.55 [1.40-1.72] | <0.001 | 21 | 1.66 [1.46-1.89] | <0.001 |
| 3: Overweight vs Normal | Death | 35 | 0.94 [0.91-0.98] | 0.0011 | 27 | 0.98 [0.94-1.01] | 0.20 |
| 4: Obese I vs Normal | Death | 31 | 1.13 [1.06-1.19] | <0.001 | 24 | 1.13 [1.04-1.21] | 0.0022 |
| 5: Obese II vs Normal | Death | 28 | 1.40 [1.28-1.54] | <0.001 | 22 | 1.39 [1.16-1.65] | <0.001 |

**Appendix Figure 1.** Spline analysis, based on the subset with BMD measures, for relationship between BMI and hip fracture, with knots at 21, 25 and 33 kg/m² in separate models for men and women, unadjusted and then adjusted for femoral neck BMD T-score. Hazard ratio for fracture (95% CI dotted lines) is versus BMI of 25 kg/m².

**BMI and hip fracture risk in men**

**
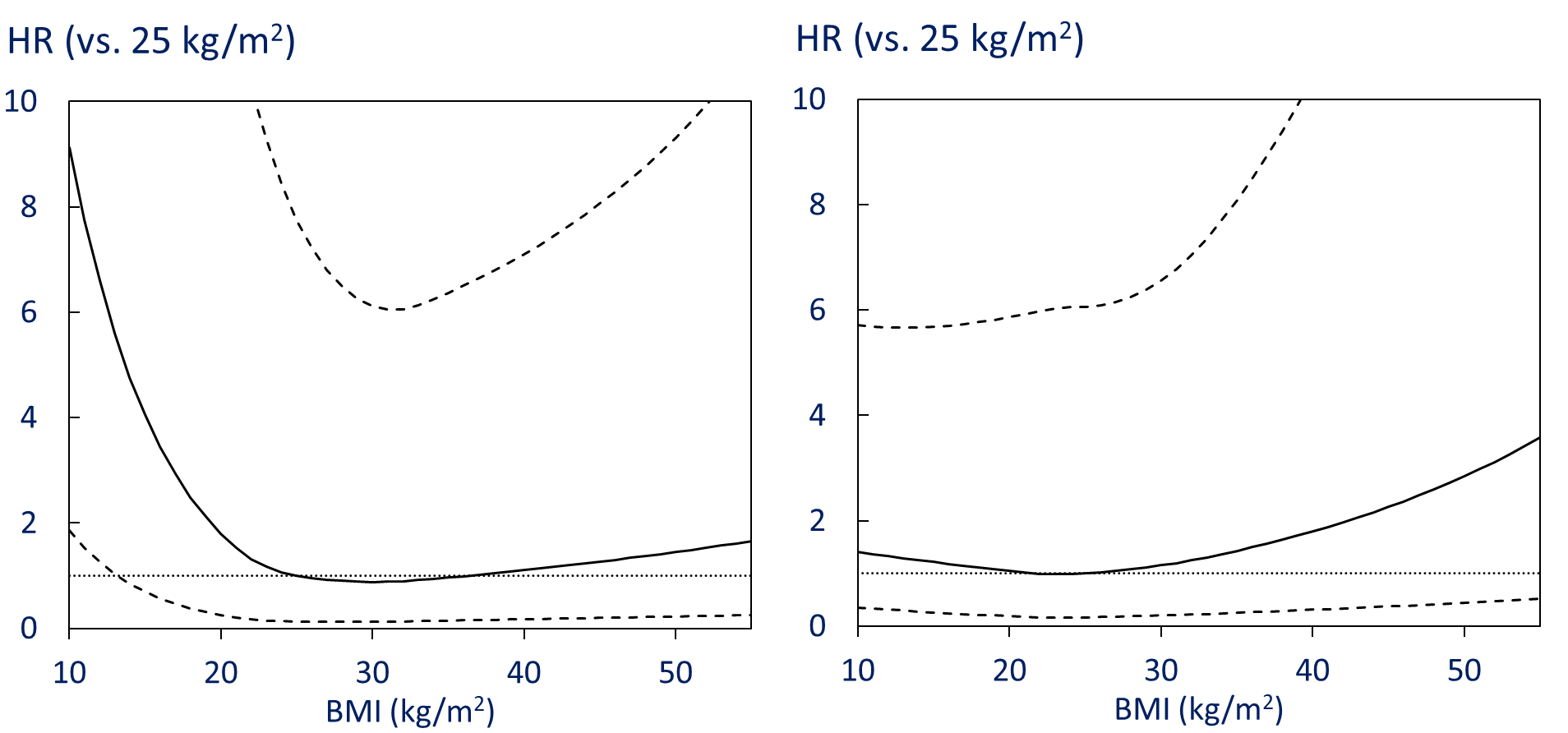
**

**BMI and hip fracture risk in women**

**
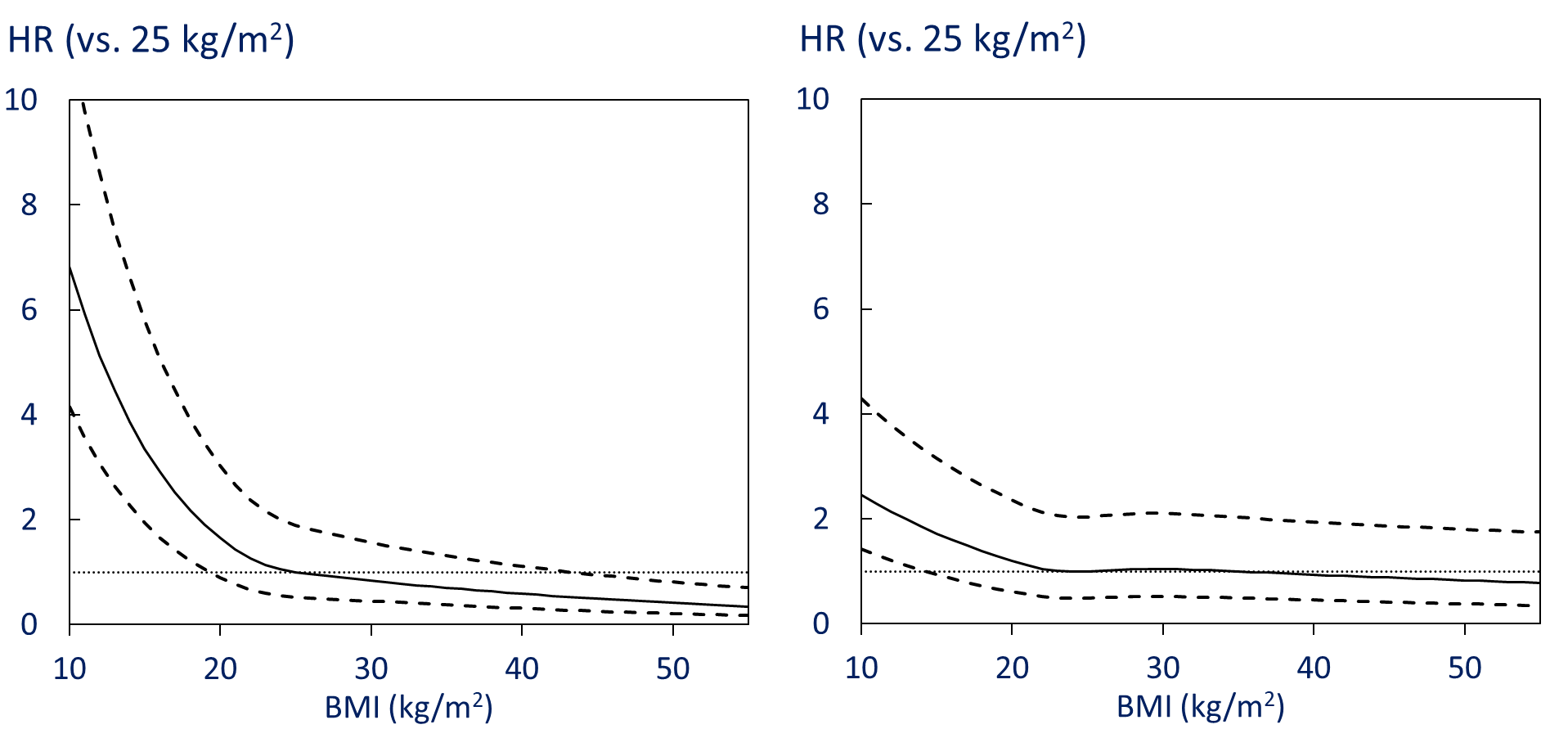
**
